# The Metabolic Syndrome among Postmenopausal Women in Gorgan

**DOI:** 10.1155/2012/953627

**Published:** 2012-02-15

**Authors:** Abdoljalal Marjani, Sedigheh Moghasemi

**Affiliations:** ^1^Department of Biochemistry and Biophysics, Biochemistry and Metabolic Disorders Research Center, Gorgan Faculty of Medicine, Golestan University of Medical Sciences, Iran; ^2^Department of Midwifery, Golestan University of Medical Sciences, Iran

## Abstract

*Introduction*. The present study aimed to assess the metabolic syndrome among postmenopausal women in Gorgan, Iran. *Materials and Methods*. The study was conducted on hundred postmenopausal women who were referred to the health centers in Gorgan. Metabolic syndrome was diagnosed using Adult Treatment Panel III (ATP III) guidelines. *Results*. The mean body mass index, waist circumference, hip, circumference waist-to-hip ratio, diastolic blood pressure, and triglyceride and fasting blood glucose levels were significantly high among postmenopausal women with metabolic syndrome, but the mean HDL-cholesterol was significantly low (*P* < 0.05). Overall prevalence of metabolic syndrome was 31%. Body mass index and waist circumference had a positive correlation with a number of metabolic syndrome factors (*P* < 0.001). Body mass index, waist circumference, and waist-to-hip ratio had a positive correlation with each other (*P* < 0.001). BMI had relatively high correlation with WC (*P* < 0.001). *Conclusions*. Our results show that postmenopausal status might be a predictor of metabolic syndrome. Low HDL-cholesterol level and high abdominal obesity are the most frequent characteristics in comparison to other metabolic components. Our study also showed some related factors of metabolic syndrome among postmenopausal women. These factors may increase cardiovascular risk among postmenopausal women with metabolic syndrome.

## 1. Introduction

The metabolic syndrome (MetS) is described by the clustering of several risk factors for cardiovascular disease (CVD) such as hypertension, dyslipidaemia, obesity (particularly central obesity), insulin resistance, and high fasting plasma glucose [1]. In 2001, The Third Report of National Cholesterol Education Program Expert Panel on Detection, Evaluation, and Treatment of High Blood Cholesterol in Adults (Adult Treatment Panel III) (ATP III) emphasized the importance of the metabolic syndrome and provided a working definition of this syndrome for the first time [2]. Some studies showed the prevalence of metabolic syndrome among postmenopausal women in China, India, and Brazil to be 33.5%, 55%, and 34.7%, respectively [3–5]. Differences in genetic background, diet, levels of physical activity, age and sex structure all influence the prevalence of both metabolic syndrome and its components [6]. Cardiovascular disease is one of the main reasons of death among women in the world [7]. The studies indicated that women aged more than 55 have a higher incidence of cardiovascular disease than younger women [8–10]. Other studies showed that there is a high prevalence of metabolic syndrome among postmenopausal women, which varies from 32.6% to 41.5% [11–13]. Study of Rossi et al. [14] demonstrated that postmenopausal women with metabolic syndrome have lower incidence of cardiovascular risk factors. The mechanism behind the role of menopausal risk factors in initiating cardiovascular disease remains unclear [15]. Some studies have shown that there is no difference in cardiovascular risk factors when comparing premenopausal with postmenopausal women [15–18]. In several studies, the incidences of metabolic syndrome among postmenopausal women were found to be increased in the world [19, 20]. Some other studies showed that there is an elevation in systolic blood pressure [21], serum total cholesterol, and triglycerides [22] and a reduction in HDL-cholesterol levels [23] among postmenopausal women. Studies in Iran showed that prevalence of the metabolic syndrome was 35–58% [24, 25]. There is no data on the prevalence of metabolic syndrome among postmenopausal women in Gorgan (South East of Caspian Sea), Iran. Therefore, it is very important to set up a study on the postmenopausal women with a risk of metabolic syndrome. The present study aimed to assess the metabolic syndrome among postmenopausal women in this area.

## 2. Subjects and Methods

This cross-sectional study was performed in the Biochemistry and Metabolic Disorders Research Center of Gorgan, Golestan province (South East of Caspian Sea, North East of Iran) in 2011. The study was conducted on hundred postmenopausal women who were referred to the different health centers in Gorgan. Postmenopausal women who had at least 1-year history of cessation of menses were included. All the included subjects provided an informed consent. At the point of study entry, all study participants were subjected to clinical and biochemical investigations. Data were collected by trained interviewers. First of all, a questionnaire was completed at each health center by trained interviewers. Demographic information is achieved by a questionnaire. The exclusion criterion was the coexistence of any other serious illness; this included having hormone replacement therapy, taking drugs such as antidiabetes and antihypertensive antilipidemic, agents and smokers. A venous blood sample was collected from all the subjects who came after 8–12 hours in the morning after an overnight fast. The samples were centrifuged for 10 minutes at 3000 rpm. The serum was used for estimating fasting blood glucose, triglycerides, total cholesterol, LDL-cholesterol, and HDL-cholesterol concentrations, by biochemical kit using spectrophotometer techniques (Model JENWAY 6105 UV/VIS) in the Biochemistry and Metabolic Disorders Research Center (School of Medicine). Postmenopausal women were considered to have metabolic syndrome if they had any three or more of the following, according to the ATP III Criteria: [2]

(A) abdominal obesity: waist Circumference >88 cm,
(B) hypertriglyceridaemia: serum triglycerides level >150 mg/dL,
(C) low HDL-cholesterol: <50 mg/dL,
(D) high blood pressure: SBP > 130 mmHg and/or DBP > 85 mmHg or on treatment for hypertension,
(E) high fasting glucose: serum glucose level > 110 mg/dL or on treatment for diabetes.

Weight was then measured, while subjects were minimally clothed without shoes, using digital scales. Height was measured in standing position without shoes using tape meter while the shoulder was in a normal position. Body mass index (BMI) was calculated as weight in kilograms divided by height in meters squared. Those with a BMI of 25.0–29.9 Kg/m^2^ were classified as overweight, whilst those with a BMI ≥ 30 Kg/m^2^ were defined as obese. Subjects with BMI greater than 45 Kg/m^2^ were considered very obese [26]. Waist circumference was measured at the point halfway between the lower border of ribs and the iliac crest in a horizontal plane [27], and hip circumference was measured at the widest level over the greater trochanters. Waist-to-hip ratio was calculated as waist circumference divided by hip circumference. Systolic and diastolic blood pressure was measured twice after 10–15 minutes resting in sitting position from the right hand. Two measurements were done all postmenopausal women at five-minute intervals and we used the average of the 2 measurements. The results were reported as percentages and mean ± SD. The statistical analysis was done with SPSS-16 version software. The results were evaluated by using Student T-test and logistic regression analysis. Statistical significance was considered at *P* < 0.05.

## 3. Results

A total of hundred postmenopausal women were studied.  Table 1 shows the baseline data of postmenopausal women with and without metabolic syndrome. The mean age of subjects was 54.12 ± 5.28 years, and the mean number of years since menopause was 5.22 ± 3.40. The mean body mass index, waist circumference, hip, waist-to-hip ratio, diastolic blood pressure, and triglyceride and fasting blood glucose levels were significantly high among postmenopausal women with metabolic syndrome, but the mean HDL-cholesterol was low (*P* < 0.05). There were no significant differences in the age, years since menopause, total cholesterol, systolic blood pressure, and LDL-cholesterol of postmenopausal women with and without metabolic syndrome.  Table 2 shows the prevalence and component of metabolic syndrome among postmenopausal women with metabolic syndrome. Overall prevalence of the metabolic syndrome among postmenopausal women was 31%. The percentages of fasting blood sugar >110 mg/dL, high density lipoprotein-cholesterol < 50 mg/dL, Triglyceride > 150 mg/dL, waist circumference > 88 cm, and systolic blood pressure > 130 mmHg/diastolic blood pressure > 85 mmHg are presented in Table 2. The percentages of these components of metabolic syndrome among postmenopausal women were 17, 30, 16, 29, and 16%, respectively.  Table 3 shows the correlation between the number of metabolic syndrome factors and body mass index, waist circumference, and waist-to-hip ratio. Linear regression analysis shows that body mass index and waist circumference had a positive correlation with the number of metabolic syndrome factors (*P* < 0.001). BMI had relatively higher correlation with number of metabolic syndrome factors (*P* < 0.001).  Table 4 shows the correlation between body mass index, waist circumference, and waist-to-hip ratio. The pearson correlation analysis shows that all indices had a positive correlation with each other (*P* < 0.001). BMI had relatively high correlation with WC (*P* < 0.001).

## 4. Discussion

In our study, we found that the mean body mass index, waist circumference, hip circumference, waist-to-hip ratio, diastolic blood pressure, and triglyceride and fasting blood glucose levels were significantly high among postmenopausal women with metabolic syndrome, but the mean HDL-cholesterol was low. Many studies address the relation of menopause with blood pressure. The study of Bengtsson showed that there is a little reduction in systolic blood pressure after menopause [28]. Some other studies showed that blood pressure is not changed with menopausal status [29]. It has been shown that there is an elevation in systolic blood pressure among postmenopausal women without any change in diastolic blood pressure [30]. Our study shows that systolic and diastolic blood pressure was high among postmenopausal women with metabolic syndrome. Our study also shows that diastolic blood pressure was significantly high among postmenopausal women with metabolic syndrome. This may suggest that diastolic blood pressure is one of the risk factors for cardiovascular disease among postmenopausal women with metabolic syndrome. Our study shows that triglycerides were significantly high among postmenopausal women with metabolic syndrome. There are different studies about the menopausal effect on triglycerides. Some studies showed no effect [15, 31, 32] while other studies showed elevations of triglyceride levels after menopause [23, 33, 34]. Our finding shows low HDL-cholesterol level among postmenopausal women with metabolic syndrome. There are some studies showing reduction and elevation of HDL-cholesterol levels following menopause [23, 35]. Most studies have shown that plasma HDL-C levels either fall slightly [36–38] or remain stable [16] with menopause. Some studies show increasing of plasma HDL-C levels after menopause in Korean and Iranian population [39, 40]. The study of Kreisberg showed that reductions in HDL-cholesterol should be taken into account as one of coronary heart disease risk factors among postmenopausal women [41]. It has been shown that there is an increase in fasting blood sugar among postmenopausal women with metabolic syndrome [42]. Our results showed a significant increase in fasting blood sugar between postmenopausal women with and without metabolic syndrome. Waist circumference was elevated among postmenopausal women with metabolic syndrome. It was also shown that postmenopausal women were overweight and obese. Body mass index and waist circumference have shown to have positive correlation with the number of metabolic syndrome factors, and also body mass index, waist circumference and waist-to-hip ratio had a positive correlation with each other. The study of Lobo showed that weight increase and obesity greatly increase prevalence of metabolic syndrome among postmenopausal women. Changes in central obesity can cause metabolism abnormality and influence health [43]. Estrogen secretion is decreased among postmenopausal women depending on metabolic alterations and resulting agglomeration of abdominal fat. It is important to reduce the risk of cardiovascular disease among postmenopausal women. It suggests that among postmenopausal women blood glucose, blood pressure, lipid profile monitoring and changing their life style can lead to weight loss by diet and sport [44]. The prevalence of metabolic syndrome among postmenopausal women is 31%. The study on postmenopausal women in Austria showed that the prevalence of metabolic syndrome was 32.6% [45] which was similar to that in our finding. In another study on postmenopausal women in Chengdu, China, the prevalence of metabolic syndrome was shown to be 37.34%, which was higher than our finding [12]. In our study, we found high prevalence of the components of metabolic syndrome among postmenopausal women. Low HDL-cholesterol and high waist circumference were the most usual factors of metabolic abnormality among postmenopausal women. Prevalence of cardiovascular diseases might be increased. This may happen with high prevalence of metabolic syndrome among postmenopausal women. The study of Deibert et al. [46] showed that prevalence of metabolic syndrome among postmenopausal women was 36.1% and also some other studies showed that menopausal women in Canada [47], Ecuador [20], and Republic of Korea [48] had prevalence of metabolic syndrome, 31%, 41.5%, and 54.6%, respectively. 

In conclusion, our results show that postmenopausal status might be a predictor of metabolic syndrome in this area. In this study, low HDL-cholesterol level and high abdominal obesity are the most frequent characteristics in comparison to other metabolic components. Our study also showed some related factors of metabolic syndrome among postmenopausal women. These factors may increase cardiovascular risk in postmenopausal women with metabolic syndrome.

## Figures and Tables

**Table 1:** Baseline data of postmenopausal women (total subjects and subjects with and without metabolic syndrome).

Parameters	Total number of postmenopausal women	Postmenopausal women with metabolic syndrome	Postmenopausal women without metabolic syndrome	*P* value
All women, no. (%)	100	31	69	
Age (years)	54.32 ± 5.26	54.51 ± 5.31	54.31 ± 5.39	0.865
Years since menopause	5.36 ± 3.38	5.0 ± 3.54	5.62 ± 3.37	0.403
BMI, kg/m^2^	30.98 ± 5.52	33.68 ± 5.40	29.77 ± 5.17	0.001
WC, cm	91.12 ± 11.70	97.98 ± 9.47	88.04 ± 11.34	<0.001
Hip circumference, cm	104.61 ± 9.23	109.03 ± 10.43	102.62 ± 7.95	0.001
WHR	0.87 ± 0.09	0.89 ± 0.07	0.84 ± 0.13	0.030
SBP, mmHg	150.23 ± 17.02	160.0 ± 20.45	135.32 ± 1.83	0.505
DBP, mmHg	78.72 ± 1.24	83.61 ± 1.10	76.52 ± 1.25	0.008
FBS, mg/dL	107.66 ± 63.53	143.45 ± 100.38	91.57 ± 24.17	0.008
TG, mg/dL	122.94 ± 91.67	171.90 ± 124.20	100.94 ± 62.0	<0.001
T-Chol, mg/dL	210.87 ± 49.28	219.71 ± 49.80	206.90 ± 48.88	0.231
HDL-Chol, mg/dL	46.49 ± 14.23	42.54 ± 8.60	48.26 ± 15.86	0.022
LDL-Chol, mg/dL	129.32 ± 40.52	128.32 ± 44.23	129.77 ± 39.07	0.870

BMI: body mass index, WC: waist circumference, WHR: waist-to-hip ratio, SBP: systolic blood pressure, DBP: diastolic blood pressure, FBS: fasting blood glucose, TG: triglyceride, T-CHOL: total cholesterol, HDL-CHOL: HDL-cholesterol, and LDL-CHOL: LDL-cholesterol.

**Table 2:** Prevalence of metabolic syndrome and the components of metabolic syndrome in postmenopausal women.

	Number	%
Metabolic syndrome	31	31
Fasting blood sugar > 110 mg/dL	17	17
High-density lipoprotein-cholesterol < 50 mg/dL	30	30
Triglyceride > 150 mg/dL	16	16
Waist circumference > 88 cm	29	29
Systolic blood pressure > 130 mmHg/diastolic blood pressure > 85 mmHg	16	16

**Table 3:** Regression analysis of number of the metabolic syndrome factors versus BMI, WC, and WHR.

	BMI	WC	WHR
*R*	0.511	0.406	0.213
*R* ^2^	0.261	0.165	0.045
Coefficient	0.053	0.024	1.667
Constant	1.623	1.039	1.926
*P*	0.003	0.024	0.251

BMI: body mass index, WC: waist circumference, and WHR: waist-to-hip ratio.

**Table 4:** Correlation between the anthropometric indices in postmenopausal women.

	BMI	WC	WHR
BMI	—	0.807*	0.363*
WC	0.807*	—	0.742*
WHR	0.363*	0.742*	—

* *P* < 0.001.

## References

[1] Miranda PJ, DeFronzo RA, Califf RM, Guyton JR (2005). Metabolic syndrome: definition, pathophysiology, and mechanisms. *American Heart Journal*.

[2] Cleeman JI (2001). Executive summary of the third report of the National Cholesterol Education Program (NCEP) expert panel on detection, evaluation, and treatment of high blood cholesterol in adults (adult treatment panel III). *Journal of the American Medical Association*.

[3] Ruan X, Jin J, Hua L, Liu Y, Wang J, Liu S (2010). The prevalence of metabolic syndrome in chinese postmenopausal women and the optimum body composition indices to predict it. *Menopause*.

[4] Shefali P, Srinivas M, Aghashe S (2010). Menopause and metabolic syndrome: a study of 498 urban women from western India. *Journal of Mid-Life*.

[5] Neto JADF, Figuerêdo ED, Barbosa JB (2010). Metabolic syndrome and menopause: cross-sectional study in gynecology clinic. *Arquivos Brasileiros de Cardiologia*.

[6] Cameron AJ, Shaw JE, Zimmet PZ (2004). The metabolic syndrome: prevalence in worldwide populations. *Endocrinology and Metabolism Clinics of North America*.

[7] Lloyd-Jones D, Adams R, Carnethon M (2009). Heart disease and stroke statistics—2009 update. A report from the American heart association statistics committee and stroke statistics subcommittee. *Circulation*.

[8] Lerner DJ, Kannel WB (1986). Patterns of coronary heart disease morbidity and mortality in the sexes: a 26-year follow-up of the Framingham population. *American Heart Journal*.

[9] Ford ES, Giles WH, Dietz WH (2002). Prevalence of the metabolic syndrome among US adults: findings from the Third National Health and Nutrition Examination Survey. *Journal of the American Medical Association*.

[10] Rosamond W, Flegal K, Friday G (2007). Heart disease and stroke statistics—2007 Update: a report from the American Heart Association Statistics Committee and Stroke Statistics Subcommittee. *Circulation*.

[11] Chedraui P, Hidalgo L, Chavez D, Morocho N, Alvarado M, Huc A (2007). Quality of life among postmenopausal Ecuadorian women participating in a metabolic syndrome screening program. *Maturitas*.

[12] Ding QF, Hayashi T, Zhang XJ (2007). Risks of CHD identified by different criteria of metabolic syndrome and related changes of adipocytokines in elderly postmenopausal women. *Journal of Diabetes and its Complications*.

[13] Ponholzer A, Temml C, Rauchenwald M, Marszalek M, Madersbacher S (2008). Is the metabolic syndrome a risk factor for female sexual dysfunction in sexually active women?. *International Journal of Impotence Research*.

[14] Rossi R, Nuzzo A, Origliani G, Modena MG (2008). Metabolic syndrome affects cardiovascular risk profile and response to treatment in hypertensive postmenopausal women. *Hypertension*.

[15] Peters HW, Westendorp ICD, Hak AE (1999). Menopausal status and risk factors for cardiovascular disease. *Journal of Internal Medicine*.

[16] Kannel WB, Hjortland MC, McNamara P, Gordon T (1976). Menopause and risk of cardiovascular disease. The Framingham study. *Annals of Internal Medicine*.

[17] Razay G, Heaton KW, Bolton CH (1992). Coronary heart disease risk factors in relation to the menopause. *Quarterly Journal of Medicine*.

[18] Hjortland MC, McNamara PM, Kannel WB (1976). Some atherogenic concomitants of menopause: the Framingham study. *American Journal of Epidemiology*.

[19] Royer M, Castelo-Branco C, Blümel JE (2007). The US National Cholesterol Education Programme Adult Treatment Panel III (NCEP ATP III): Prevalence of the metabolic syndrome in postmenopausal Latin American women. *Climacteric*.

[20] Hidalgo LA, Chedraui PA, Morocho N, Alvarado M, Chavez D, Huc A (2006). The metabolic syndrome among postmenopausal women in Ecuador. *Gynecological Endocrinology*.

[21] Lindquist O (1982). Intraindividual changes of blood pressure, serum lipids, and body weight in relation to menstrual status: results from a prospective population study of women in Goteborg, Sweden. *Preventive Medicine*.

[22] Bonithon-Kopp C, Scarabin PY, Darne B, Malmejac A, Guize L (1990). Menopause-related changes in lipoproteins and some other cardiovascular risk factors. *International Journal of Epidemiology*.

[23] Jensen J, Nilas L, Christiansen C (1990). Influence of menopause on serum lipids and lipoproteins. *Maturitas*.

[24] Azizi F, Salehi P, Etemadi A, Zahedi-Asl S (2003). Prevalence of metabolic syndrome in an urban population: Tehran Lipid and Glucose Study. *Diabetes Research and Clinical Practice*.

[25] Sarrafzadegan N, Kelishadi R, Baghaei A (2008). Metabolic syndrome: an emerging public health problem in Iranian Women: Isfahan Healthy Heart Program. *International Journal of Cardiology*.

[26] World Health Organization (1998). *Prevention and Management of the Global Epidemic of Obesity. Report of the WHO Consultation on Obesity*.

[27] Dalton M, Cameron AJ, Zimmet PZ (2003). Waist circumference, waist-hip ratio and body mass index and their correlation with cardiovascular disease risk factors in Australian adults. *Journal of Internal Medicine*.

[28] Bengtsson C, Lindquist O (1979). Menopausal effects on risk factors for ischaemic heart disease. *Maturitas*.

[29] Meade TW, Haines AP, Imeson JD (1983). Menopausal status and haemostatic variables. *Lancet*.

[30] Weiss NS (1972). Relationship of menopause to serum cholesterol and arterial blood pressure: the United States’health examination survey of adults. *American Journal of Epidemiology*.

[31] Davis CE, Pajak A, Rywik S (1994). Natural menopause and cardiovascular disease risk factors: The Poland and US collaborative study on cardiovascular disease epidemiology. *Annals of Epidemiology*.

[32] Casiglia E, D’Este D, Ginocchio G (1996). Lack of influence of menopause on blood pressure and cardiovascular risk profile: a 16-year longitudinal study concerning a cohort of 568 women. *Journal of Hypertension*.

[33] Reardon MF, Nestel PJ, Craig IH, Harper RW (1985). Lipoprotein predictors of the severity of coronary artery disease in men and women. *Circulation*.

[34] Bergman S, Siegert G, Wahrburg V (1997). Influence of menopause and life style factors on high-density lipoproteins in middle aged women. *Journal of North American Menopause Society*.

[35] Do KA, Green A, Guthrie JR, Dudley EC, Burger HG, Dennerstein L (2000). Longitudinal study of risk factors for coronary heart disease across the menopausal transition. *American Journal of Epidemiology*.

[36] Matthews KA, Meilahn E, Kuller LH, Kelsey SF, Caggiula AW, Wing RR (1989). Menopause and risk factors for coronary heart disease. *New England Journal of Medicine*.

[37] Torng PL, Su TC, Sung FC (2000). Effects of menopause and obesity on lipid profiles in middle-aged Taiwanese women: the Chin-Shan Community Cardiovascular Cohort Study. *Atherosclerosis*.

[38] Torng PL, Su TC, Sung FC (2002). Effects of menopause on intraindividual changes in serum lipids, blood pressure, and body weight—the Chin-Shan community cardiovascular cohort study. *Atherosclerosis*.

[39] Azizi F, Ainy E (2003). Coronary heart disease risk factors and menopause: a study in 1980 Tehranian women, the Tehran Lipid and Glucose Study. *Climacteric*.

[40] Kim CJ, Kim TH, Ryu WS, Ryoo UH (2000). Influence of menopause on high density lipoprotein-cholesterol and lipids. *Journal of Korean Medical Science*.

[41] Kreisberg RA, Kasim S (1987). Cholesterol metabolism and aging. *The American Journal of Medicine*.

[42] Walton C, Godsland IF, Proudler AJ, Wynn V, Stevenson JC (1993). The effects of the menopause on insulin sensitivity, secretion and elimination in non-obese, healthy women. *European Journal of Clinical Investigation*.

[43] Lobo RA (2008). Metabolic syndrome after menopause and the role of hormones. *Maturitas*.

[44] Heidari R, Sadeghi M, Talaei M, Rabiei K, Mohammadifard N, Sarrafzadegan N (2010). Metabolic syndrome in menopausal transition: Isfahan Healthy Heart Program, a population based study. *Diabetology and Metabolic Syndrome*.

[45] Ponholzer A, Temml C, Rauchenwald M, Marszalek M, Madersbacher S (2008). Is the metabolic syndrome a risk factor for female sexual dysfunction in sexually active women?. *International Journal of Impotence Research*.

[46] Deibert P, König D, Vitolins MZ (2007). Effect of a weight loss intervention on anthropometric measures and metabolic risk factors in pre- versus postmenopausal women. *Nutrition Journal*.

[47] Piché MÈ, Weisnagel SJ, Corneau L, Nadeau A, Bergeron J, Lemieux S (2006). The WHO and NCEP/ATPIII definitions of the metabolic syndrome in postmenopausal women: are they so different?. *Metabolic Syndrome and Related Disorders*.

[48] Hee MK, Park J, So YR, Jongoh KIM (2007). The effect of menopause on the metabolic syndrome among Korean wwomen: the Korean National Health and Nutrition Examination Survey, 2001. *Diabetes Care*.

